# A partnership between Māori healing and psychiatry in Aotearoa New Zealand

**DOI:** 10.1192/bji.2022.25

**Published:** 2023-05

**Authors:** Wiremu NiaNia, Allister Bush

**Affiliations:** 1 Tohunga, Turuki Health Care, Auckland, New Zealand; 2 Consultant Child and Adolescent Psychiatrist, Te Whare Mārie, Māori Mental Health Service, Te Whatu Ora, Porirua, New Zealand. Email: allister.bush@mhaids.health.nz

**Keywords:** Māori, healing, Indigenous, partnership, psychiatry

## Abstract

This paper describes an example of Māori healing and psychiatry working together in an Indigenous mental health context in Aotearoa (New Zealand). Each author outlines their perspectives on the context and the partnership. The case of a Māori teenager with pseudo-seizures and voice-hearing is described to illustrate the partnership in action.

## Wiremu

My name is Wiremu NiaNia and I work as a Māori healer. As you may know, Māori are the Indigenous people of Aotearoa and inhabited our country for many generations prior to the arrival of Europeans from the late 1700s onwards. My tribal affiliations are with Tuhoe, Ngāti Tuwharetoa and Ngāti Kahungunu. I grew up in a family adoption with my kuia (my adopted grandmother) and we spoke te reo Māori (Māori language) in our whānau (family). It was only when I went to school that I had to speak English.

I was identified by my kuia as having a fine attunement to Te Ao Wairua (the spiritual realm) at a young age. She was a healer and guided me in how to live with this awareness and how to use it to help others.

Among principles I use in my work, the most important is that spirituality is the foundation of everything else, illustrated in this proverb:

‘I hangaia tātou e te kaihanga, kia hīkoi tātou te hīkoi tāngata.’

‘We were created by God to journey this earth as humans. First and foremost we are spiritual beings experiencing a human existence.’^1^

When I meet with an individual or family, naturally they bring their own faith and cultural beliefs. I don't mind what their religious traditions or spiritual background may be. I consider God as my source and without that connection I can do nothing. Any healing comes from that source.

However, I am mindful of history. While I respect Christianity, many churches got tied up with colonisation. Biblical teachings were used to justify land confiscations from my people.^1^ Christianity silenced Māori understandings about wairua (spirituality) and healing, so Indigenous knowledge was eroded. For example, my kuia taught that spiritual experiences are common and the spiritual realm is part and parcel of everyday life. These experiences can be positive or could be distressing. We as Māori have many ways to understand and address them. In contrast, according to Christian teachings, unseen voices or visions are often viewed as ‘demonic’. As a result, people may feel afraid of or disempowered by such experiences. This is frequently unhelpful. I offer people my Māori views on how to make sense of such puzzling experiences.^1^ I want them to have tools to understand and manage their experiences. Awareness and knowledge are empowering and can help people take charge of their own healing.

When people are hearing voices or seeing things that are unexplained, they may worry they have a psychiatric problem. I acknowledge that other perspectives can be helpful. Allister, a psychiatrist, and I have been working together over the past 18 years, since we met at Te Whare Mārie, a Māori mental health service. Even though I left that workplace some years ago, we continue teaching and writing together about this work.^1–3^ With our ongoing exchange of ideas, we have developed a working relationship that allows space for both Māori healing and psychiatry perspectives. We find that families and individuals appreciate accessing both types of expertise.

Most of my practice is focused on spiritual healing. However, alongside wairua (spiritual well-being) Māori healing also encompasses tinana (physical), hinengaro (psychological) and whānau (relational) aspects and more broadly can include rongoa (herbal treatments) and mirimiri (massage treatments).^1,4^ In addition, the healing quality of water was always highly valued by my ancestors. Our healing traditions have been handed down in families from generation to generation.

For Allister and me to work together effectively, we are mindful of the history between our cultures and professions. Psychiatry in New Zealand was part of the process of colonisation that side-lined Māori healing and separated us as Māori from our own cultures and identities. After European settlers arrived in Aotearoa, there was systematic undermining of Māori healing practices by colonial authorities, culminating in the Tohunga Suppression Act of 1907, which outlawed Māori healing activities.^1^ This suppression of Indigenous approaches to healing followed the same pattern observed in other parts of the world where European powers colonised Indigenous peoples.^1^

Therefore, a partnership between Indigenous healing and psychiatry is not simple. It requires trust, mutual respect and acknowledgement of the historical background, or the same mistakes from the past could interfere with our work together. I chose Tātaihono as a name to describe this type of partnership. Hono refers to joining and Tātaihono is about a bond that requires commitment and a genuine connection. I have previously explained:

‘Tātaihono can be about reparation, reconciliation, collaboration and connection. It is about a binding together, a kind of spiritual binding that gives unity and strength.’^1^

To foster such a relationship, humility on both sides is necessary. That was a value that my kuia insisted we live by: ‘Me whakaiti koe i a koe, ma ētahi koe e whakanui’. That means: ‘You must always be the least, and let others raise you up’. Over many years I learned the hard way that this discipline is necessary for spiritual protection for myself and those around me. Without this I could not assist others as a healer and it is unlikely that Allister and I could work together effectively.

## Allister

My name is Allister Bush and I am a Pākehā (New Zealand European) child and adolescent psychiatrist working at Te Whare Mārie. My ancestors came from Ireland and England in the latter part of the 1800s. I had little exposure to Māori culture and values until my early adult years, but following my medical school education and during my training in psychiatry I had an extended placement at Te Whare Mārie. When I started my current position, I met Wiremu, who was employed as a cultural therapist. After some time, I became curious about his approach as I was hearing from families and other Māori clinicians about positive outcomes. He subsequently agreed that I could sit in and observe consultations with him.

### Case example

An early occasion when I had this opportunity related to a 17-year-old Māori man we will call George.^1,2^ I first met George and his mother after he was referred on to our service some months after the sudden onset of puzzling and distressing seizure-like symptoms, in which he suffered bilateral jerking movements of his upper limbs without losing consciousness. He had diabetes of several years’ duration and had been otherwise well. He had started smoking cannabis and drinking alcohol 6 months before. The onset of his symptoms occurred while out one night with friends. They were alarmed by his behaviour and called an ambulance. He was then assessed in the local emergency department, where doctors concluded his seizure-like episodes were not likely to be a form of epilepsy. Computed tomography head scan, electroencephalography and blood tests were all normal. Within a few days, George began to hear a voice that criticised him and told him to kill himself. He described feeling a negative presence next to him and believed that the voice emanated from that presence. He described it as ‘like a 24-hour a day bully’. The seizure episodes and the voice continued over a 5-month period, including during 6 weeks of antipsychotic treatment with risperidone, prescribed by the psychiatrist he met initially.

At my first meeting with George and his mother, he told me that since this risperidone treatment the voice was less loud and less frequent but still there. I discovered that the family originated from a traditional Māori tribal area and were familiar with Māori spiritual and cultural values and beliefs. George revealed that he had previously had a range of spiritual experiences, for example describing being aware of the supportive presence of a deceased favourite uncle next to him. At the burial of a teenage neighbour, he recalled seeing a vision of the teenager, who said ‘Tell my family I am OK’. These experiences were considered normal in George's family. However, his recent experiences were disturbing and had a markedly different quality. Owing to the family's Māori background and familiarity with spiritual experiences, I described Wiremu's role as a cultural therapist and offered an appointment with him, which they accepted.

## Wiremu

When I first met George, I felt a spiritual presence with him that was not nice. I could feel it was trying to control him and remain hidden. During the session, when I spoke about this, George started to have a convulsion. After he had a few moments to recover from that, I explained to him that the entity was causing this in order to maintain control. I explained Māori cultural concepts to George and his mother to give them more understanding. I explained about his mana, his spiritual authority. This gives him the ability to be selective about what entities he allows in his personal space. I encouraged him to take that authority back.

I asked about any recent conflict in the extended family and his mother explained that there had been a severe rift 6 months before that I came to believe was an important part of why George was spiritually vulnerable at that time. I advised George not to smoke cannabis anymore. I told him this substance can open up a spiritual doorway in young people who are spiritually aware, which he might find difficult to close; it could therefore render him vulnerable to many unpleasant spiritual experiences. Finally, I sought his permission to offer a karakia (prayer or incantation) for him and his whānau (family), which he accepted.

## Allister

Following this session, George cancelled our follow-up appointment 4 weeks later. When seen 2 months after the session, he reported no more seizure-like episodes, no more voices and he had only once felt like the negative entity was nearby. He stopped his risperidone 1 week after meeting Wiremu, because of weight gain and because he accepted Wiremu's explanation. He remained free of any psychiatric or neurological conditions 12 years later.

Detailed discussion about this case, from both Māori healing and psychiatric perspectives, is beyond the scope of this article, but has been explored elsewhere.^1,2^ In brief, possible psychiatric explanations I considered for his predicament included psychosis, perhaps secondary to his substance use; a dissociative disorder; or an organic problem such as temporal lobe epilepsy. Wiremu's view was that George had a Māori spiritual problem. He considered that a Māori spiritual solution was likely to resolve it. Together we were able to discuss both perspectives with the family. In George's case, his pattern of symptoms did not fit diagnostic criteria for a major psychotic disorder or epilepsy, and the time frame of the resolution of his symptoms was more consistent with Wiremu's diagnosis and intervention than with a dissociative disorder. In addition, it was Wiremu's explanation that resonated most with the family's Māori world-views.

## Conclusions

Partnerships between Indigenous healers and psychiatrists are possible and can offer Indigenous people access to mainstream psychiatric care alongside healing methods indigenous to their culture of origin. Although there are many barriers to such professional relationships, including the histories of colonial suppression of Indigenous knowledge, this article outlines some conditions under which working together in this way can be beneficial, and provides a case illustration to show this collaboration in practice.

## Data Availability

Data availability is not applicable to this article as no new data were created or analysed in this study.
